# Supplementary material for: Functional and dynamic polymerization of the ALS-linked protein TDP-43 antagonizes its pathologic aggregation
Source: Nat Commun. 2017 Jun 29;8:45. doi: 10.1038/s41467-017-00062-0 (PMC5491494; doi:10.1038/s41467-017-00062-0)
Supplement: Supplementary file 1 — Supplementary Information [file 41467_2017_62_MOESM1_ESM.pdf]

File name: Supplementary Information
Description: Supplementary figures and supplementary tables.

File name: Peer review file
Description:

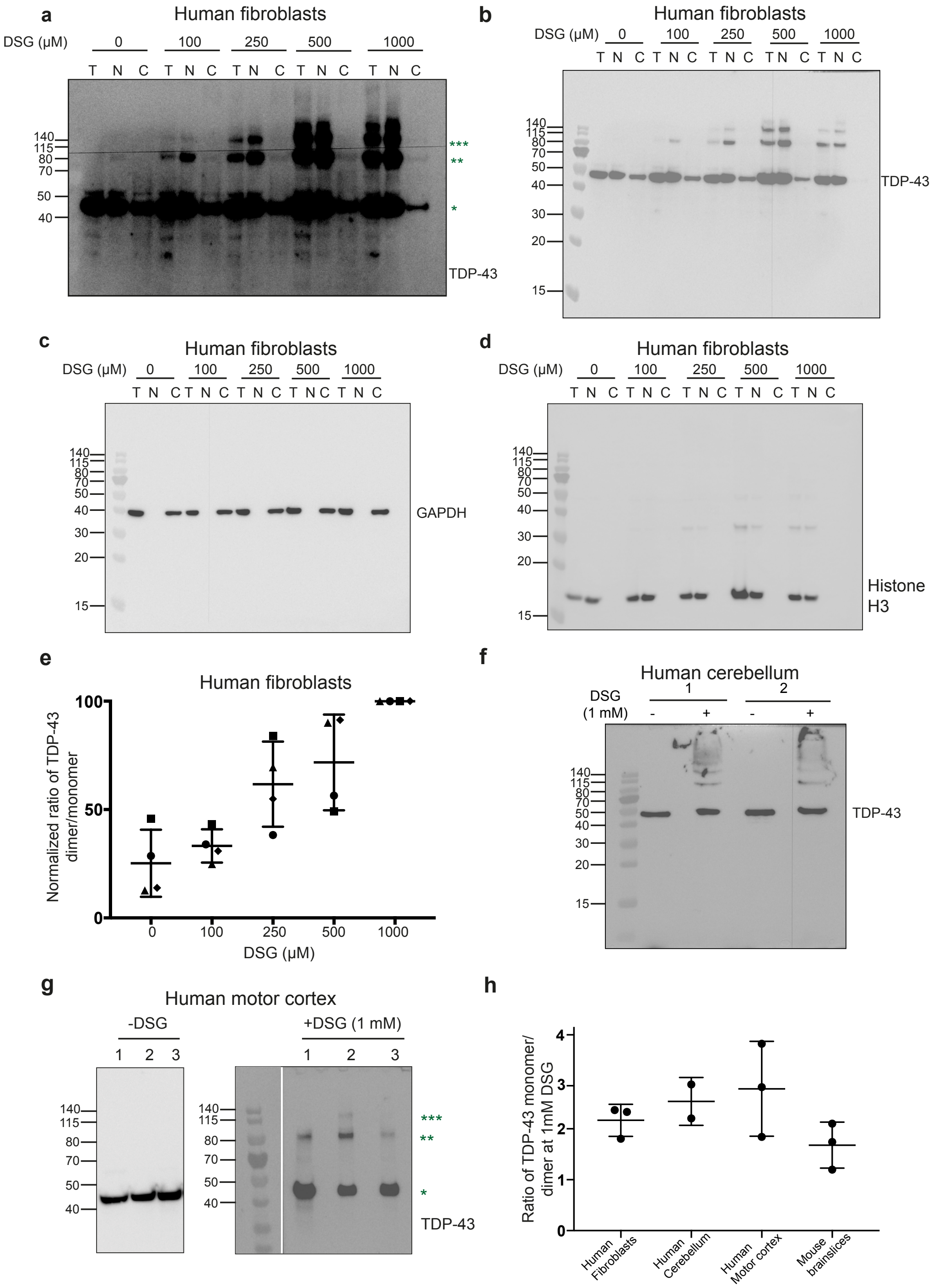

Supplementary Figure 1 – TDP-43 oligomerization in human fibroblasts, human brain tissue and mouse brain slices. (a) Overexposure of immunoblot corresponding to **Fig. 1a** shows presence of trace amounts of high molecular weight TDP-43 in the cytosolic fractions.

(b-d) Full anti-TDP-43 (b), anti-GAPDH (b) and anti-Histone H3 (c) immunoblots corresponding to **Fig. 1a** with the molecular weight markers. Total lysates (T), nuclear (N) or cytoplasmic (C) fractions were obtained from human fibroblasts, cross-linked with increasing concentrations of DSG.

(e) Quantification of relative amounts of dimeric to monomeric TDP-43 with increasing DSG concentration in human fibroblasts. Each symbol corresponds to one independent experiment (n = 4). Ratios of dimeric to monomeric TDP-43 were normalized to highest DSG concentration (1000 μM) for each experiment.

(f) Full anti-TDP-43 immunoblot corresponding to **Fig. 1b** of human cerebellum samples, with molecular weight markers.

(g) Anti-TDP-43 immunoblot of human motor cortex samples in the absence (-DSG, left panel) or presence of 1 mM DSG (right panel). Green asterisks mark high molecular weight TDP-43 positive oligomers resulting from DSG cross-linking (right panel).

(h) Quantification of monomer to dimer TDP-43 bands across various biological samples cross-linked at 1 mM DSG suggests comparable ratios of monomeric to dimeric TDP-43.

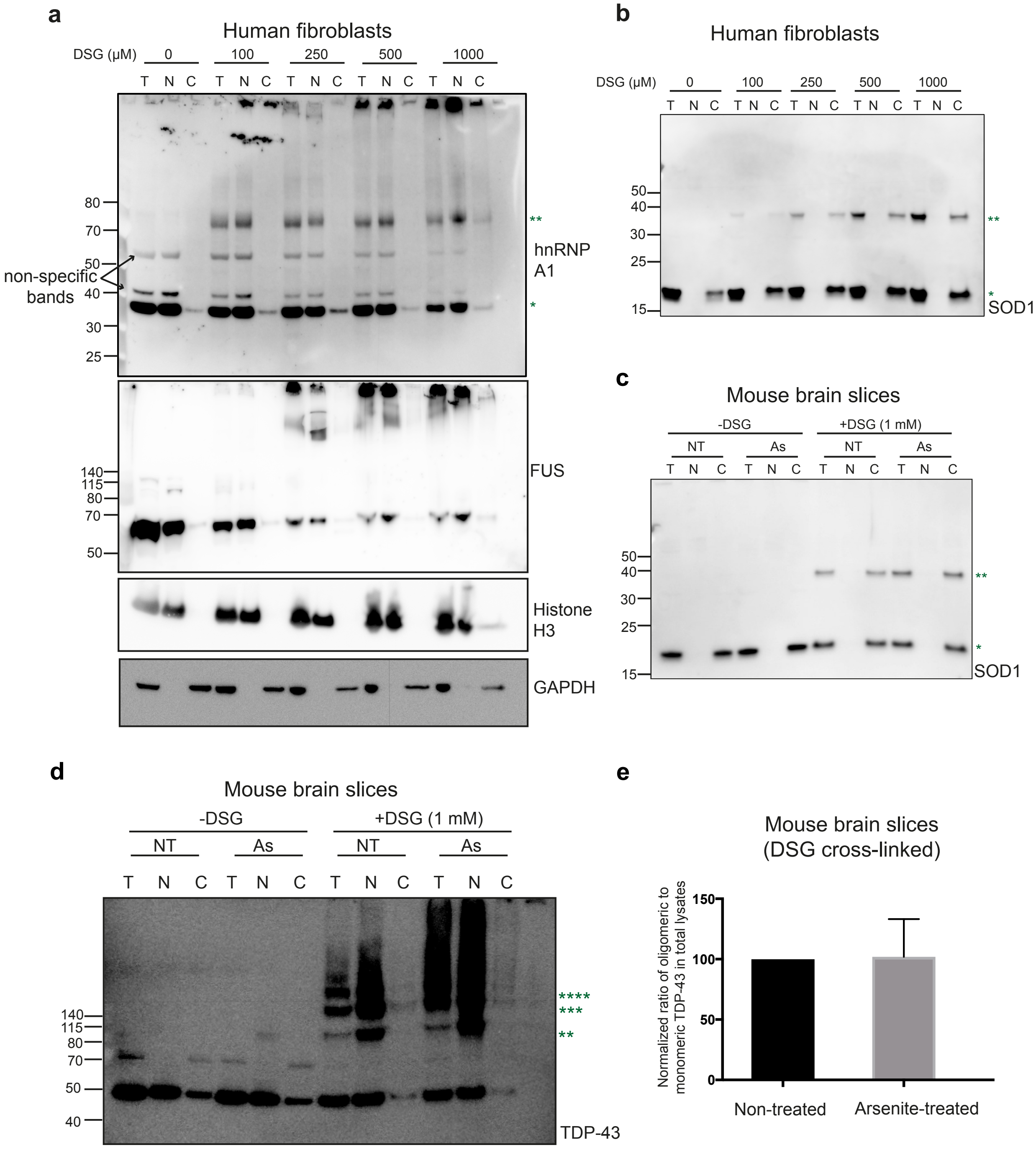

Supplementary Figure 2 – Specificity of DSG-mediated cross-linking in human cells and tissues.

(a) Immunoblots (from 4-12% denaturing polyacrylamide gels) of human fibroblast fractions obtained upon incubation with increasing concentration of DSG cross-linker followed by nucleo-cytoplasmic fractionation. In the upper panel, in addition to monomeric hnRNP A1, dimeric protein bands (marked with green asterisks) are detected with anti-human hnRNP A1 antibody at increasing concentrations of DSG, predominantly in the nuclear fraction (N) and in total cell lysates (T). The second panel shows the immunoblot with anti-human FUS antibody, where high molecular weight bands are retained in the stacking gel. Lower panels show immunoblots for the cytoplasmic marker GAPDH or the nuclear marker histone H3. Immunoblots are representative of three independent experiments.

(b-c) Full anti-SOD1 immunoblots of human fibroblasts (b) or mouse brain slices (c) showing specific DSG-mediated cross-linking of cytoplasmic SOD1 homodimers (monomers and dimers marked by green asterisks). Total lysates (T), nuclear (N) and cytoplasmic (C) fractions are shown and both monomers and dimers of SOD1 are found primarily in the cytoplasm, as expected.

(d) Overexposure of immunoblot corresponding to **Fig. 1e** shows the presence of trace amounts of high molecular weight TDP-43 in the cytosolic fractions.

(e) Normalized ratio of oligomeric to monomeric TDP-43 in non-treated and Arsenite treated DSG cross-linked mouse brain slices quantified from two independent experiments.

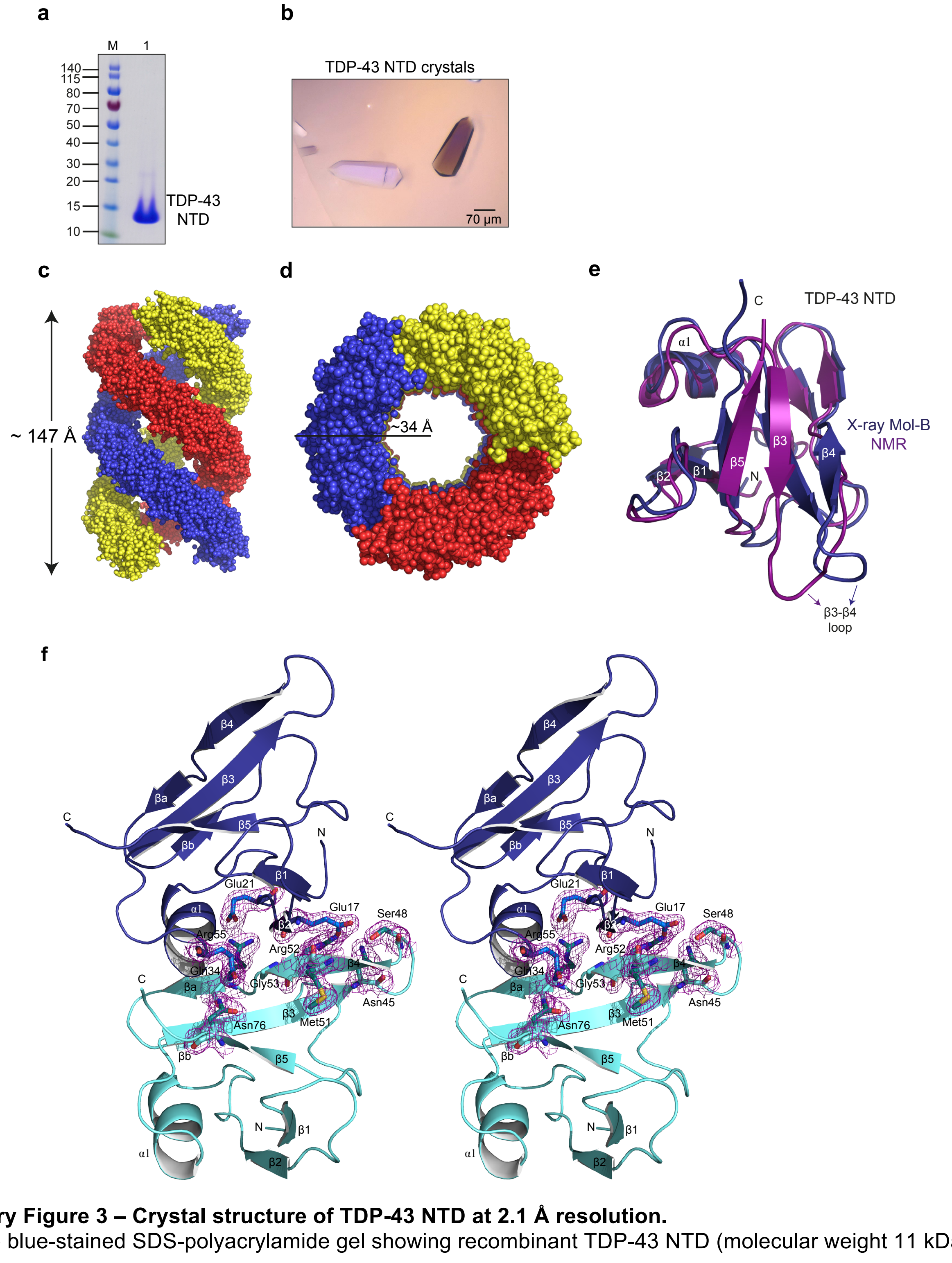

Supplementary Figure 3 – Crystal structure of TDP-43 NTD at 2.1 Å resolution.

(a) Coomassie blue-stained SDS-polyacrylamide gel showing recombinant TDP-43 NTD (molecular weight 11 kDa) purified to homogeneity.

(b) Examples of TDP-43 NTD crystals obtained after micro-seeding seen in cross-polarized light. Crystals sized up to 200 µm in length and 70 µm in diameter were obtained.

(c-d) Super-helical arrangement of TDP-43 NTD helical filaments in the crystals seen from the side (c) or the top (d) with the approximate dimensions indicated. The three helical filaments forming the super-helical arrangement are color-coded each in yellow, red and blue.

(e) Superimposition of previously reported TDP-43 NTD monomeric NMR structure (in magenta, PDB ID 2N4P) on one molecule (Mol-B) of the asymmetric unit of TDP-43 NTD crystal structure (in blue).

(f) Wall-eyed stereo view of the 2Fo-Fc map at contour level of 1.0 σ (in magenta) for the amino acids at the interface between two TDP-43 NTD monomers (one in blue, the other in cyan) in the asymmetric unit. The side chains of interacting residues are shown as sticks and the electron density in magenta.

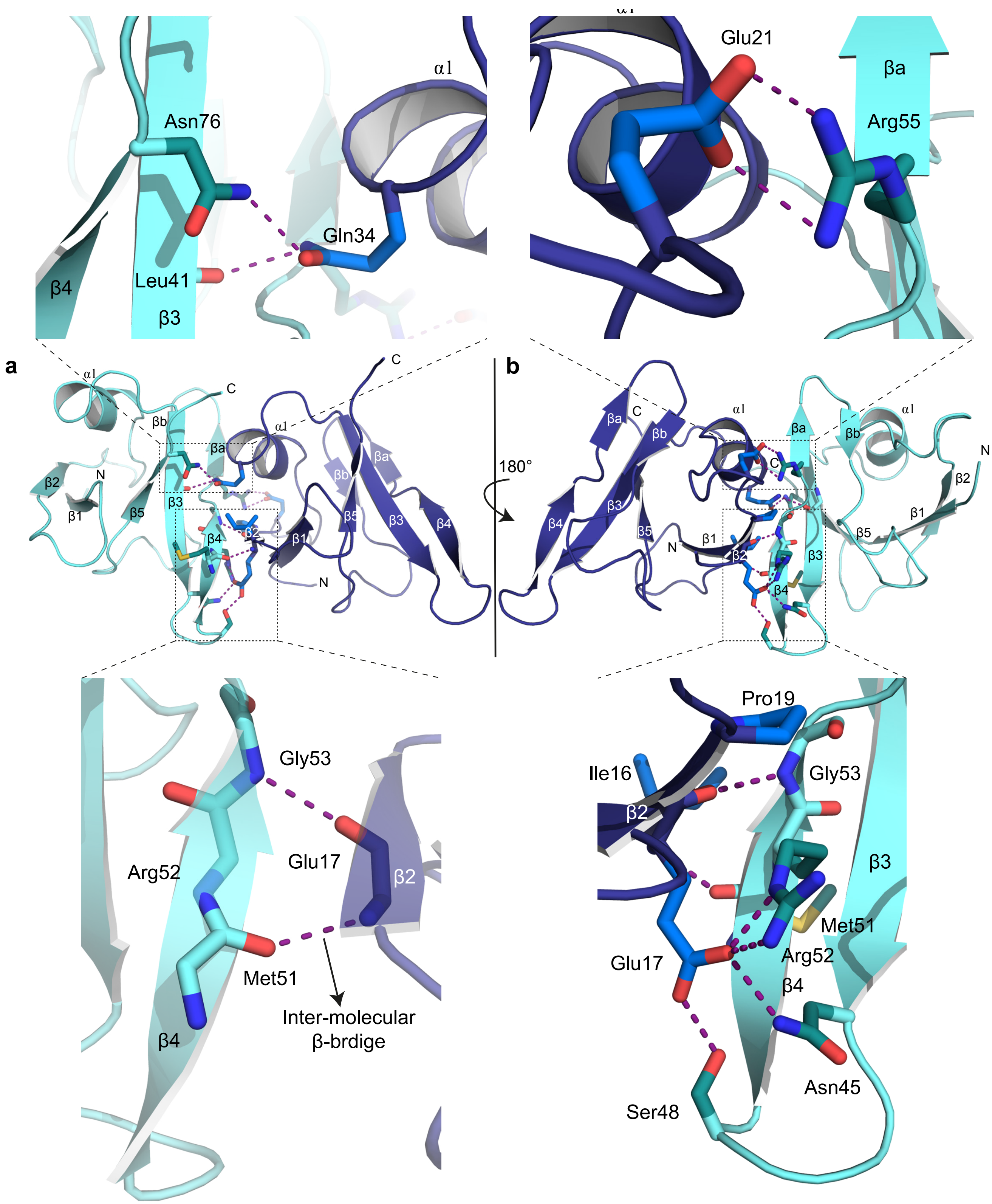

Supplementary Figure 4 – Inter-molecular interactions between TDP-43 NTD monomers in the crystal structure.
(a-b) Two TDP-43 NTD molecules in the asymmetric unit are colored in blue and cyan and shown in cartoon representation with secondary structure elements labeled (middle panel). Side chains at the interface of two molecules are shown in stick representation and inter-molecular hydrogen bonds are shown as dashed lines in magenta. Zoom-in views of the interacting side chains at the interface of two monomers corresponding to the region in black dashed boxes are shown above and below the full structures.

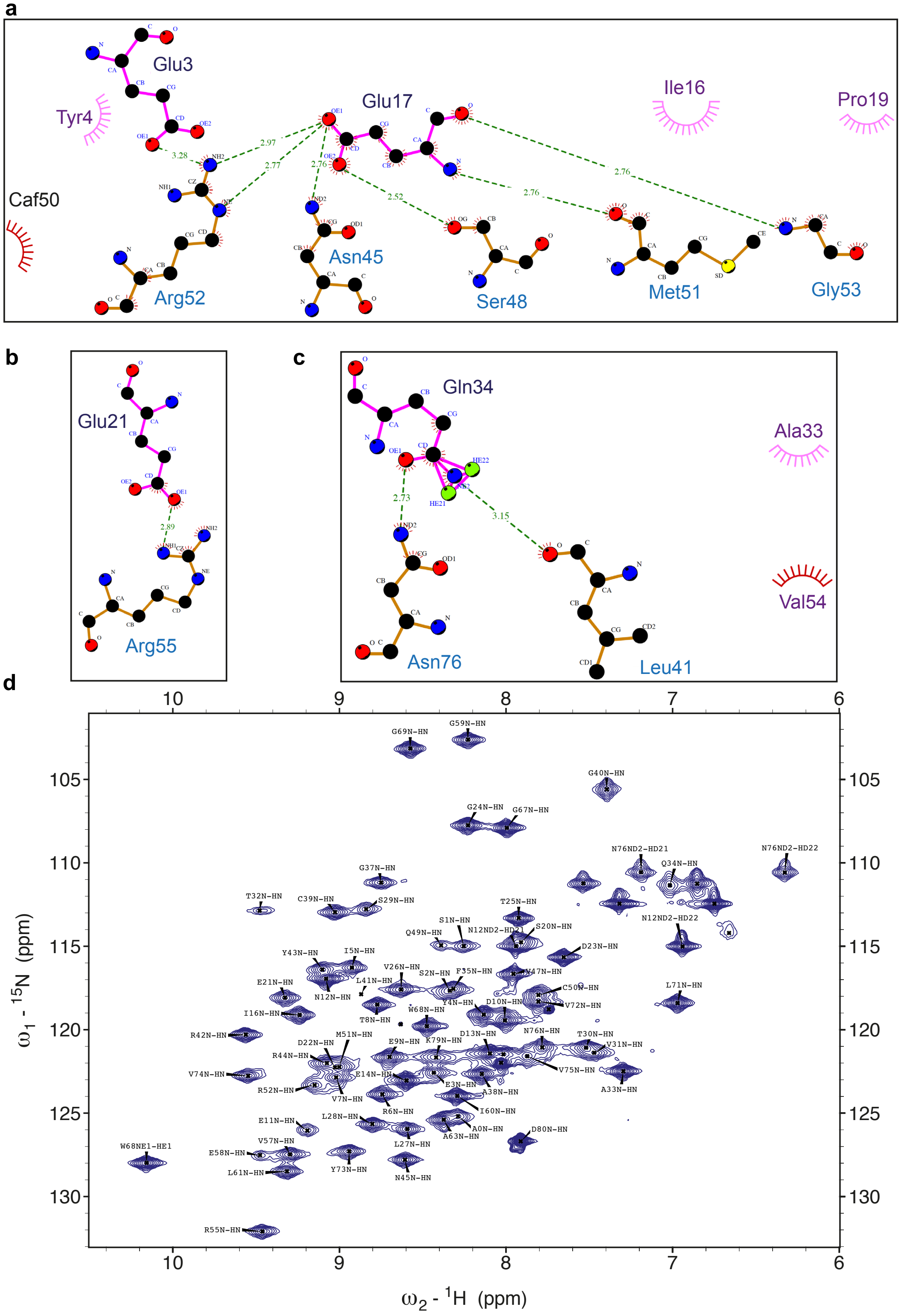

Supplementary Figure 5 – Inter-molecular interactions in TDP-43 NTD crystal structure and TDP-43 NTD resonance assignments in solution by NMR spectroscopy.

(a-c) Details of inter-molecular interactions at the interface of two TDP-43 NTD molecules in the asymmetric unit generated by Ligplot. Amino acids from the head region (Asn76, Leu41, Arg55, Arg52, Met51, Ser48, Asn45 and Gly53) are each labeled in cyan while residues from the tail region (Gln34, Glu21 and Glu17) are each labeled in dark blue. Side chains of amino acids are shown in ball and stick representation and individual atoms are labeled. Inter-molecular hydrogen bonds are depicted as dashed green lines and labeled with corresponding distance. Amino acids contributing to hydrophobic interactions are depicted in magenta.

(d) 2D ^{1}H-^{15}N HSQC spectrum showing backbone resonance assignments of wild type human TDP-43 NTD at 40 °C.

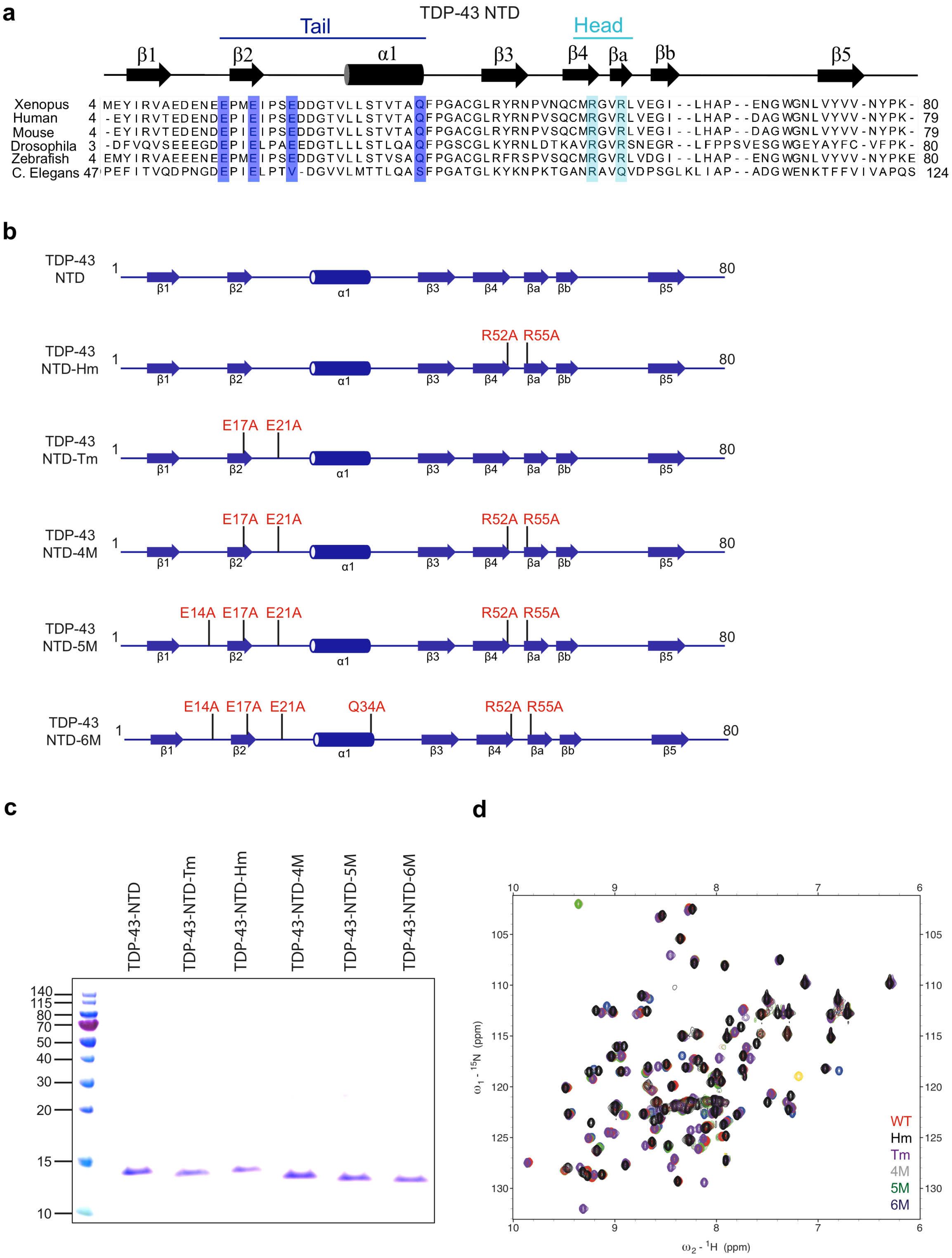

Supplementary Figure 6 – Expression and purification of TDP-43 NTD mutants disrupting TDP-43 oligomerization interface.

(a) Multiple sequence alignment of TDP-43 NTD from various species. Sequence alignment was done by T-coffee and analyzed in Jalview. The main residues involved in interaction at the interface of two molecules (cyan in head and blue in tail region) are highlighted and show conservation across species. Secondary structure elements are depicted above the sequence alignment.

(b) Secondary structure of TDP-43 NTD and the position of various amino acid substitutions (labeled in red) present in the oligomerization mutants tested in this study with their respective label shown on the left.

(c) Coomassie blue-stained SDS-polyacrylamide gel showing the purified recombinant human wild type or oligomerization mutants of TDP-43 NTD produced.

(d) Overlay of 2D ^{1}H-^{15}N HSQC spectra of wild type TDP-43 NTD and the corresponding oligomerization mutants shows that the NTD domain folding is preserved in the presence of the amino acid substitutions in mutant proteins.

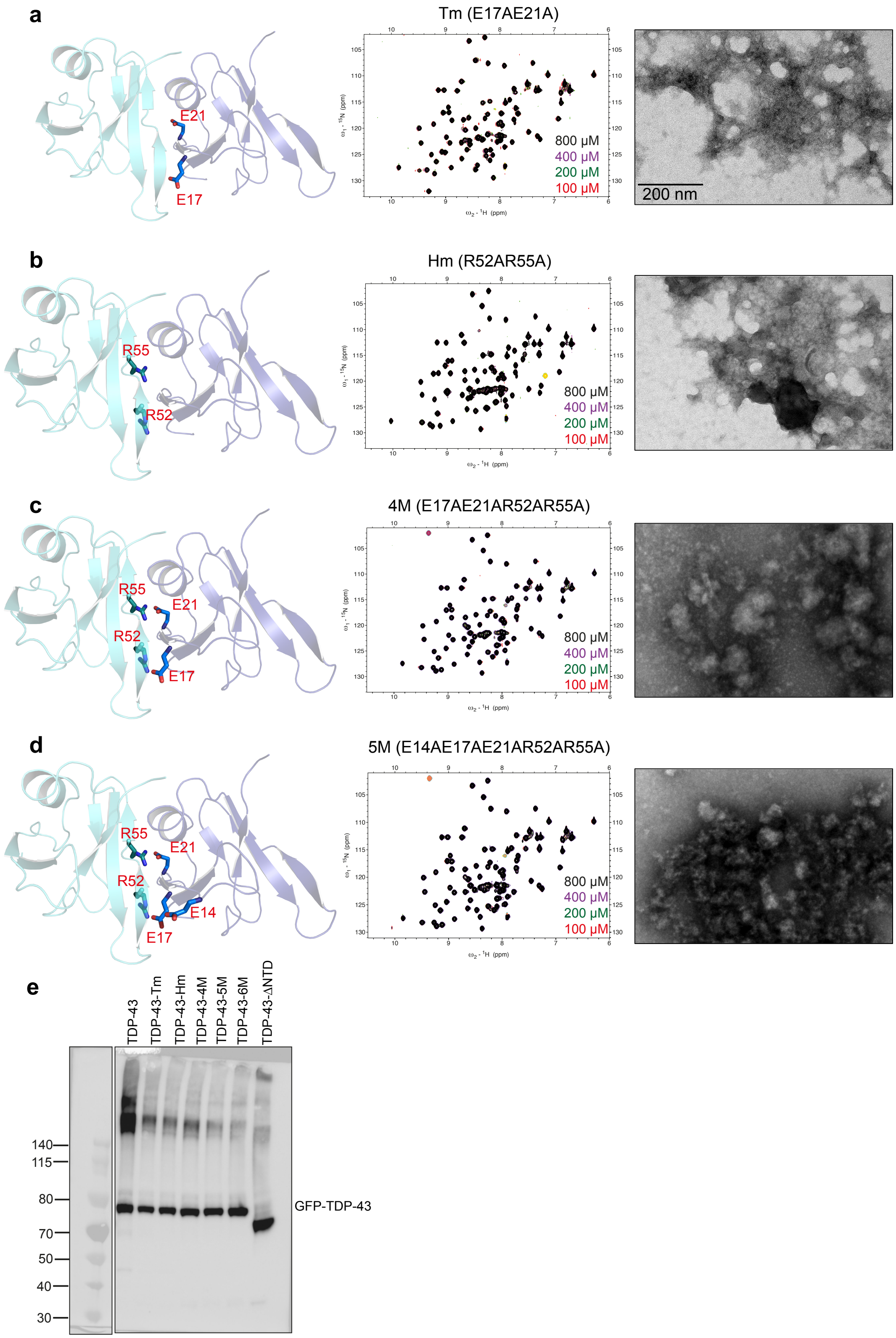

Supplementary Figure 7 – Amino acid substitutions at the intermolecular interaction surface abrogate TDP-43 NTD oligomerization *in vitro*.

(a-b) Double substitutions in the tail (Tm) or the head (Hm) region (E17AE21A (a) and R52AR55A (b), respectively), as well as in both regions (4M (c) and 5M (d), respectively) are shown on the crystal structure with side chains labeled (left panel). All mutant proteins fail to oligomerize as shown by unchanged 2D ^{1}H-^{15}N HSQC NMR spectra overlaid for increasing protein concentrations color-coded as indicated (middle panel). Right panel shows the TEM images of the corresponding mutant proteins at 100 μM, which do not form fibrillar oligomers (right panel).

(e) Full anti-TDP-43 immunoblot corresponding to **Fig. 4f** of human fibroblast samples, with molecular weight markers.

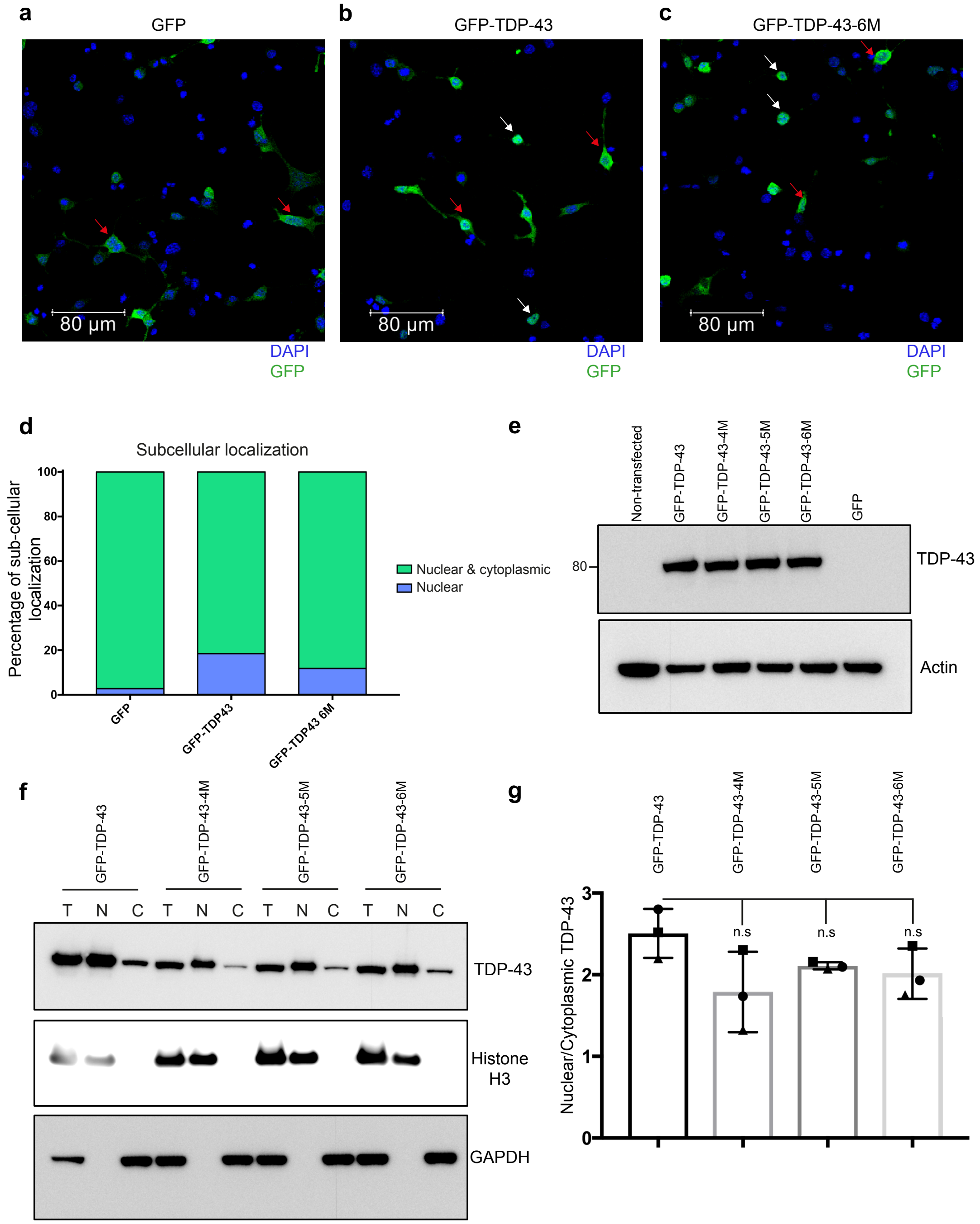

Supplementary Figure 8 – Analysis of subcellular localization of oligomerization defective TDP-43 mutants.
(a-c) Representative confocal microscope images of NSC-34 cells, overexpressing GFP (a), wild type GFP-TDP-43 (b) or oligomerization mutant GFP-TDP-43-6M (c). Fixed and permeabilized cells were stained with DAPI (blue) and anti-GFP antibody (green). Examples of cells showing only nuclear TDP-43 localization (indicated by white arrows) or both nucleo-cytoplasmic distribution (red arrows), is shown.
(d) For quantification, counting was conducted manually on 20x images acquired by confocal imaging. Approximately 750 GFP-positive cells were counted for each condition. Relative percent values of subcellular localization are shown for only nuclear (blue, co-localizing with the DAPI-positive nuclei) and the remaining nucleo-cytoplasmic (green) distribution of TDP-43.
(e) Immunoblots of total cell lysates obtained from NSC-34 cells, transiently transfected wild type and oligomerization mutant TDP-43 show similar protein expression levels. Actin is shown as a loading control.
(f) Immunoblot of total (T), nuclear (N) and cytoplasmic (C) fractions obtained following transient transfections of NSC-34 cells with wild type and oligomerization mutant TDP-43. Lower panels show immunoblots for cytoplasmic marker GAPDH or nuclear marker histone H3. Immunoblots are representative of three independent experiments.
(g) Quantification of ratio of nuclear to cytoplasmic TDP-43 in wild type and oligomerization mutants in transiently transfected NSC-34 cells from three independent experiments. The nucleo-cytoplasmic TDP-43 ratios are similar in wild type and oligomerization mutants.

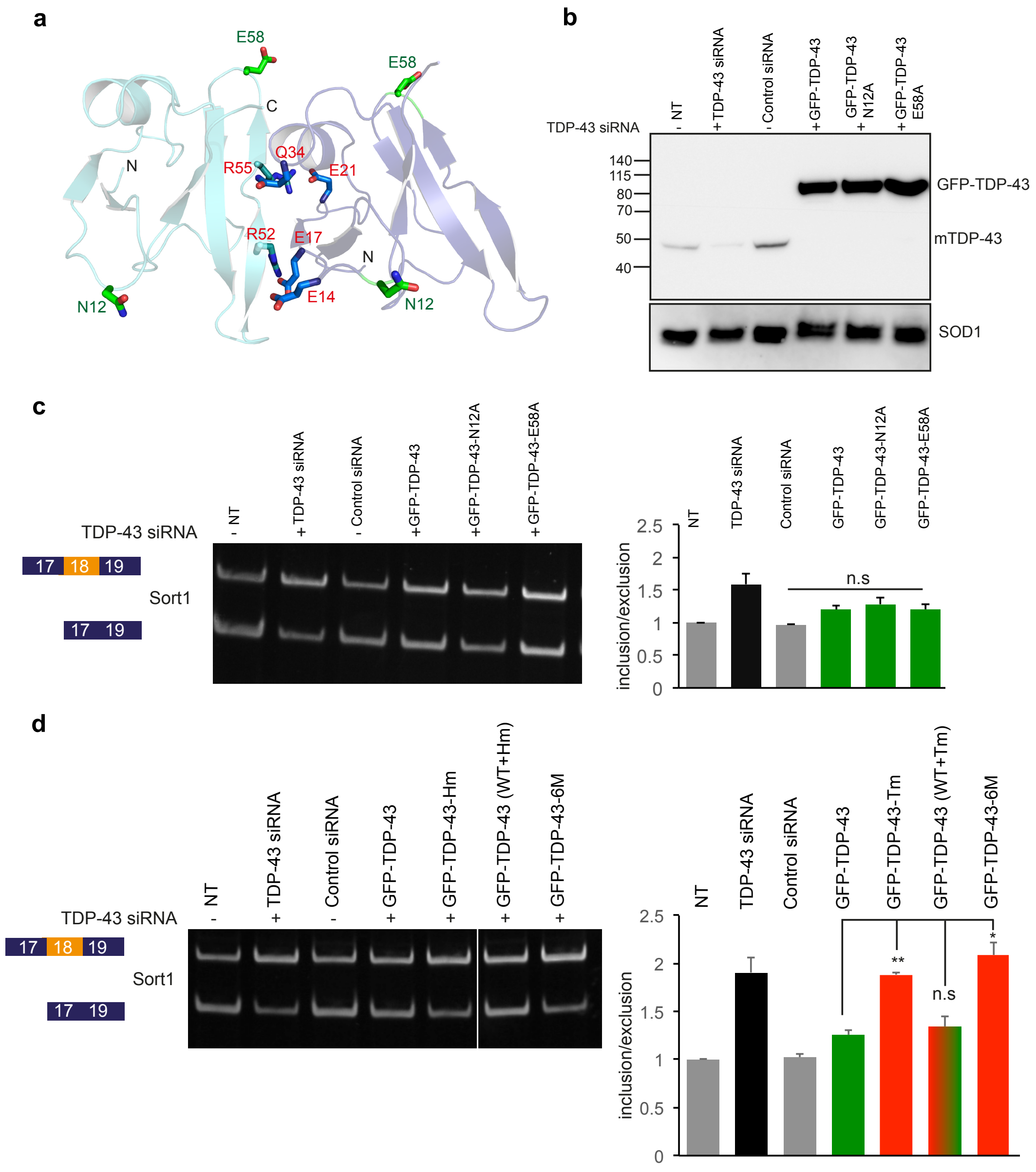

Supplementary Figure 9 – TDP-43 oligomerization is essential for its role in RNA metabolism.

(a) Position of the six amino acids that were substituted to abolish oligomerization are shown on the cartoon of TDP-43 NTD crystal structure with side chains shown as sticks and labeled in red. Two other point mutations (N12A in β1-β2 loop and E58A in βa-βb hairpin loop) that do not affect TDP-43 oligomerization are shown in green.

(**b**) Immunoblot (from 12% denaturing polyacrylamide gel) using an antibody against mouse/human TDP-43 demonstrating specific down-regulation of endogenous mouse TDP-43 and overexpression of RNAi resistant GFP-tagged wild type and mutant human TDP-43 (GFP-TDP-43-N12A/E58A) in mouse NSC-34 cells (upper panel). SOD1 is used as protein loading control (lower panel). Immunoblots are representative of three independent experiments.

(c) Rescue of *Sortilin1* splicing pattern upon endogenous TDP-43 knockdown and simultaneous expression of TDP-43 harboring NTD mutations that do not affect oligomerization (N12A and E58A). Semi-quantitative RT-PCR analysis of *Sortilin1* alternative splicing regulated by TDP-43 is shown. Left panel depicts alternatively spliced exons (orange) flanked with their constitutive exons in blue boxes. Middle panel shows the representative polyacrylamide gel images of semi-quantitative RT-PCR product. Right panel shows the quantification of splicing changes plotted as the ratio of exon inclusion/exclusion (on y-axis) against different conditions (on x-axis) averaged from three independent experiments and normalized to the ratio obtained for not treated (NT) cells that is arbitrarily set to 1. The expression of wild-type TDP43 (green bars) rescues the changes in splicing caused by down-regulation of endogenous mouse TDP-43 (TDP-43 si-RNA, black bars). The effect of each TDP-43 mutant that does not affect oligomerization (N12A and E58A) is non-significant compared to wild type protein.

(d) *Sortilin1* splicing assay (as described above) using TDP-43 with mutations in only one interaction interface (Tm, E17AE21A). As seen in quantifications, Tm (red bar, similar to 6M) is unable to rescue the splicing defect caused by endogenous TDP-43 depletion. Co-transfection of Tm together with the wild type protein (red-green) can rescue the splicing defect similar to wild type TDP-43 alone (green+red bar).

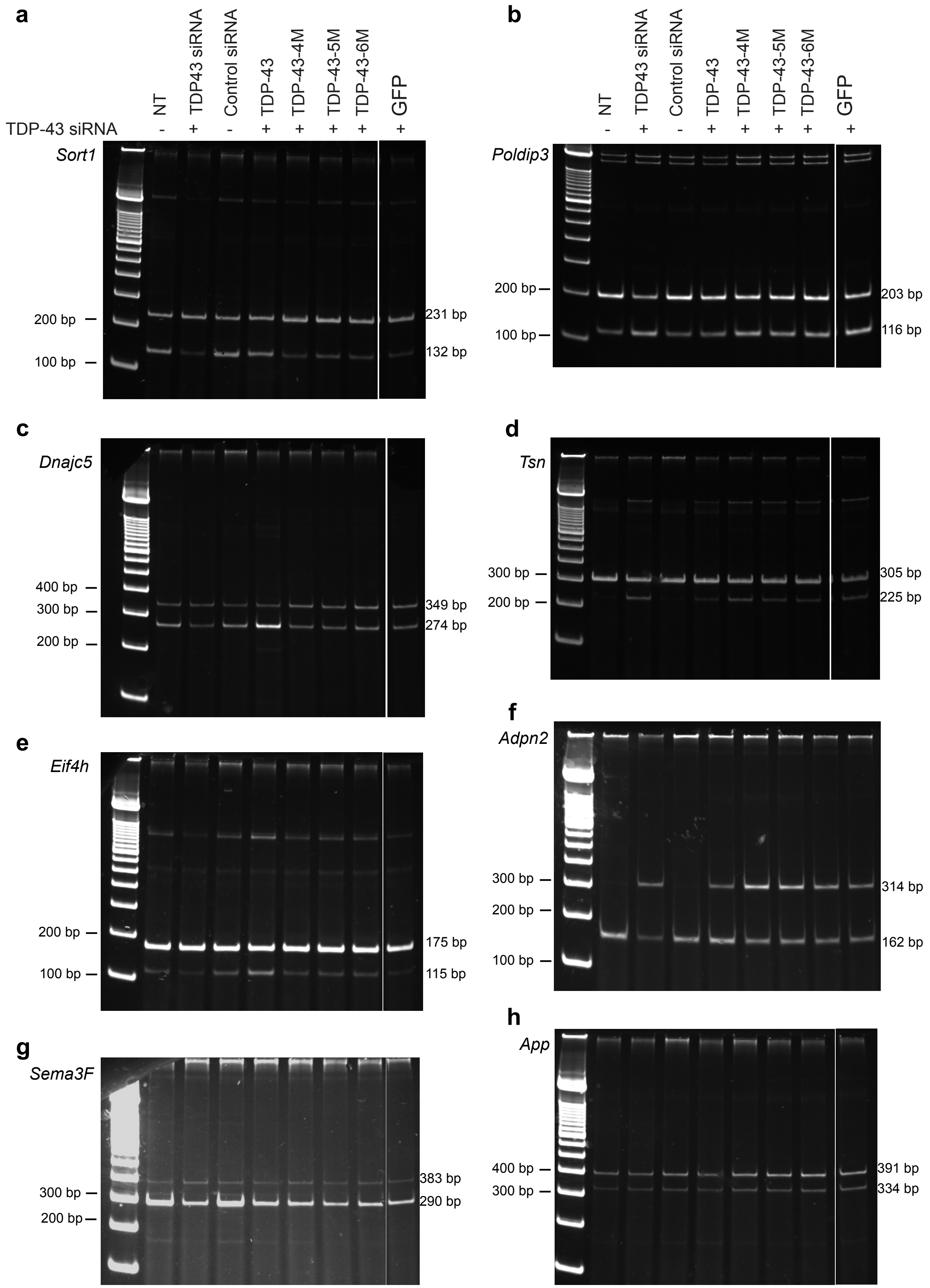

Supplementary Figure 10 – Full semi-quantitative RT-PCR gels to analyze the role of TDP-43 oligomerization in alternative splicing.

(a-h) Full 10% polyacrylamide gels corresponding to **Fig. 5c**. Size of expected PCR products corresponding to the various two alternative splice isoforms or the cryptic exon inclusion is indicated on the right of the gel for each RNA analyzed.

a

Complement with GFP_{1-9}		Complement with GFP_{1-10}	
T_{10}-TDP43<br>T_{11}-TDP43	+	T_{10}-TDP43<br>T_{11}-TDP43	+
TDP43-T_{10}<br>TDP43-T_{11}	-	TDP43-T_{10}<br>TDP43-T_{11}	+
T_{10}-TDP43<br>TDP43-T_{11}	-	T_{10}-TDP-43<br>TDP43-T_{11}	+
TDP43-T_{10}<br>T_{11}-TDP43	-	TDP-43-T_{10}<br>T_{11}-TDP43	+

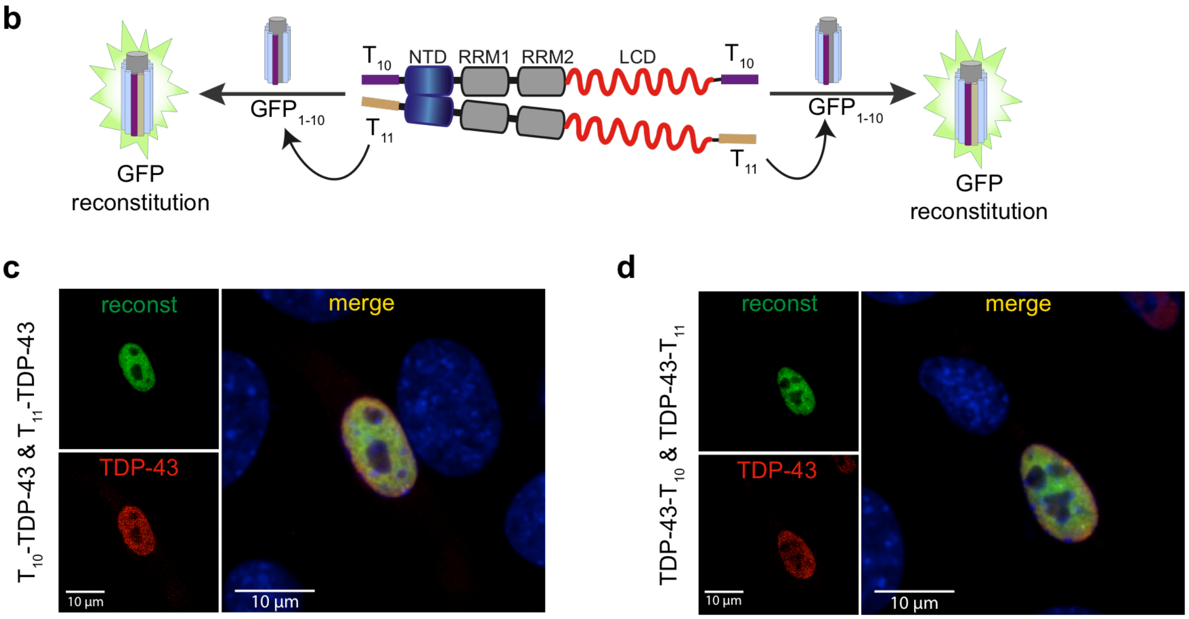

Supplementary Figure 11 – NTD-mediated TDP-43 oligomerization impedes inter-molecular LCD interactions.
(a) Table summarizing the results of fluorescence reconstitution in tripartite and dipartite GFP complementation experiments with various combinations of N- or C-terminally tagged T_{10}/T_{11} TDP-43 fusion constructs.
(b) Schematic of dipartite GFP complementation experiment. TDP-43 was tagged either at N- or C-terminus with 10th (T_{10}) and 11th (T_{11}) β-strand of GFP (shown in magenta and light yellow). A successful complementation results in fluorescence reconstitution with GFP_{1-10} molecule (β-strands 1-9 shown in blue and α-helix in grey).
(c-d) N- (c) or C-terminally (d) tagged T_{10}- and T_{11}- TDP-43 were co-transfected with GFP_{1-10}. Both N- (c) and C- (d) terminally T_{11}-tagged TDP-43 successfully complement GFP_{1-10} resulting in GFP reconstitution and relative fluorescence (in green), as seen in confocal microscope images counter-stained with an anti-TDP-43 antibody (red). The merge of the two images is zoomed-in and shown on right including DAPI-staining of the nuclei (blue).

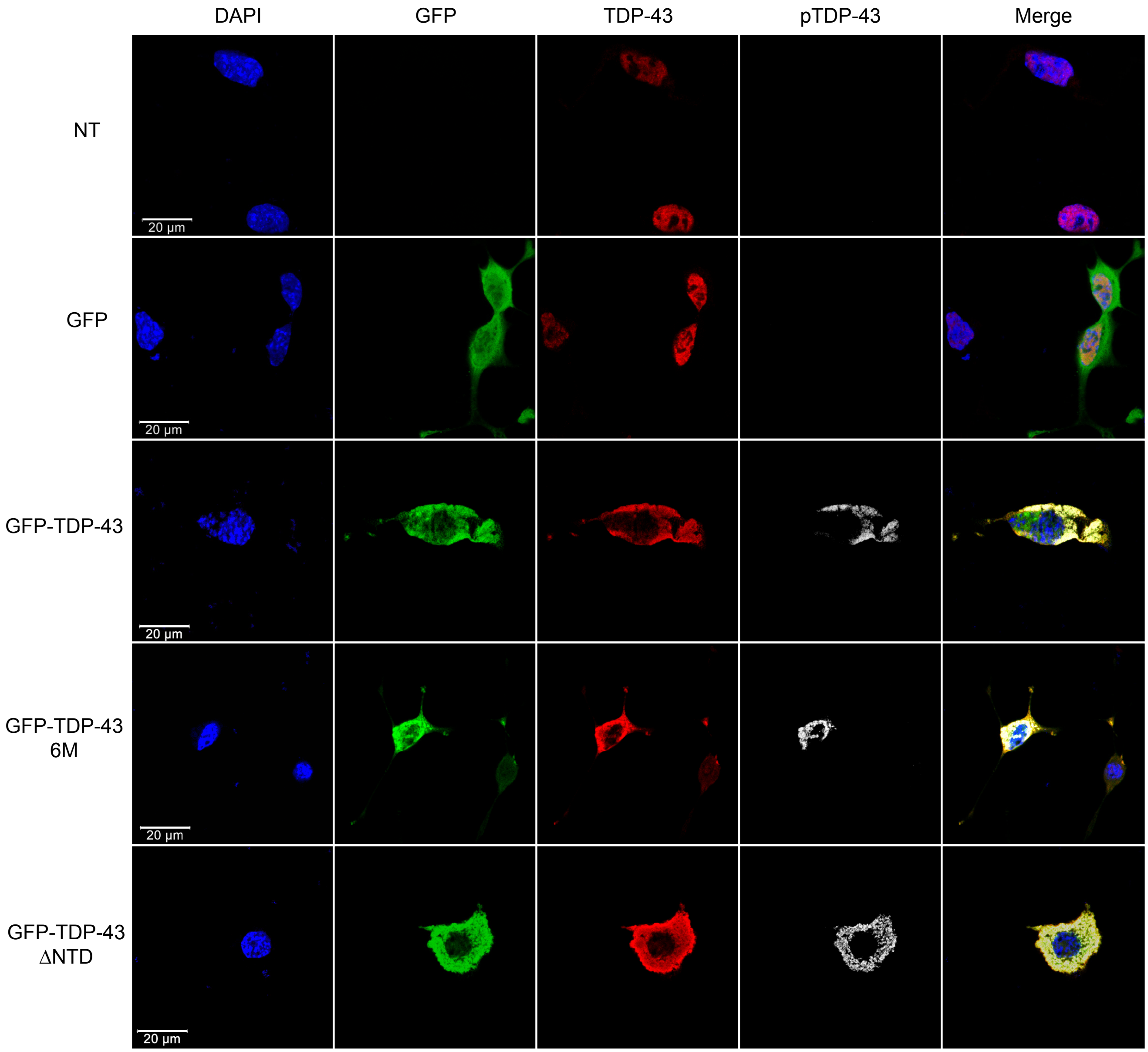

Supplementary Figure 12 – Quantitative analysis of phosphorylated TDP-43 in wild type and oligomerization mutants.

(a) Representative images of mouse NSC-34 cells transiently transfected with GFP-tagged wild type or the indicated oligomerization mutants of TDP-43. Overexpressed TDP-43 was detected with an anti-GFP antibody (green), total TDP-43 was stained using a monoclonal anti-TDP-43 antibody (red), and phosphorylated TDP-43 (grey) was detected with a specific anti-phosphoTDP-43 antibody (see **Supplementary Table 1** for the details relating to the antibody used). Nuclei were stained with DAPI (blue). Merge stained images are each shown on the last column on the right. Not transfected (NT) or GFP-transfected (GFP) cells served as controls.

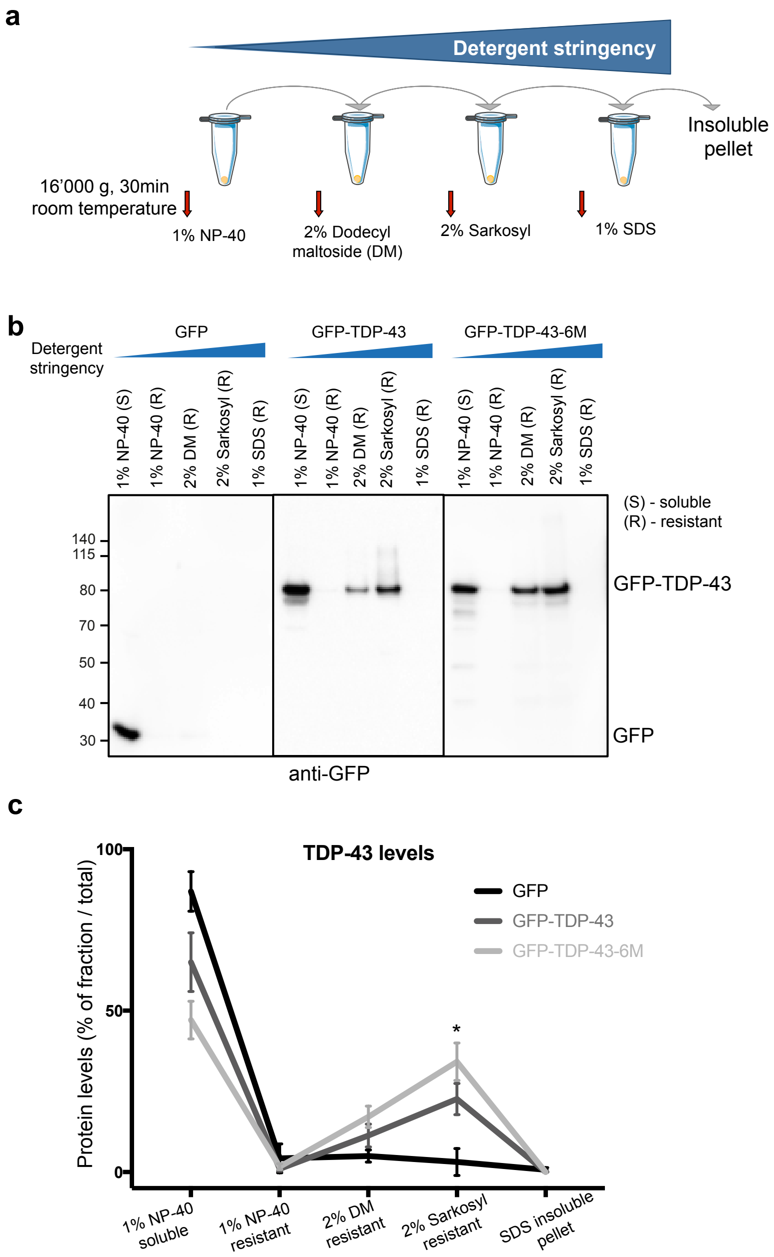

Supplementary Figure 13 - Quantitative analysis of TDP-43 insolubility in wild type and oligomerization mutants.
(a) Scheme depicting sequential insolubility assay with increasing detergent stringency.
(b) Representative immunoblots of soluble protein fractions obtained at each step of SIA detected with anti-GFP antibody.
(c) Quantifications of protein fractions obtained at each step of SIA obtained from three independent experiments. Detergent soluble and resistant TDP-43 fractions plotted as a percentage of total TDP-43 for various steps of SIA, shows enhanced insolubility of oligomerization mutants compared to wild type.

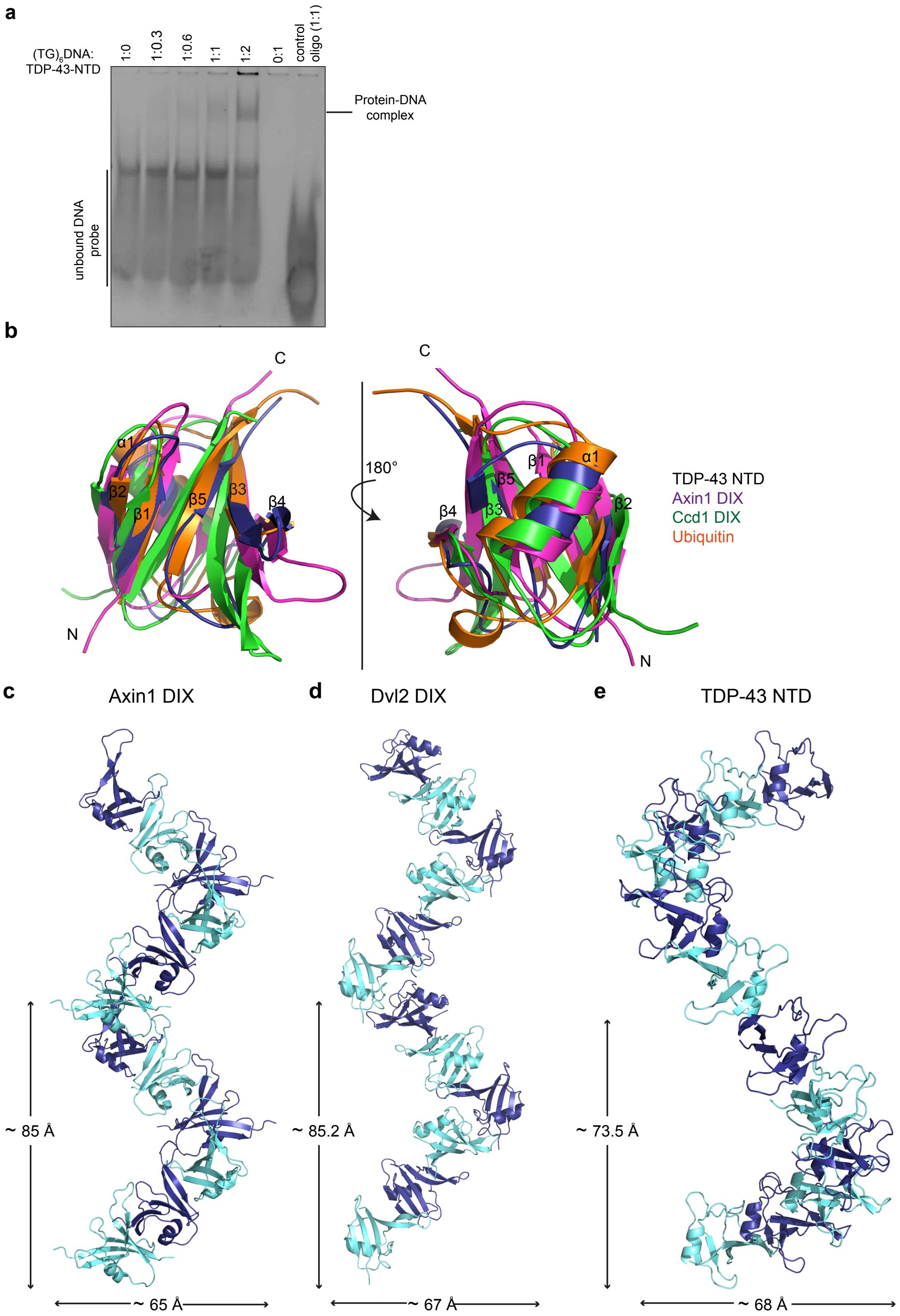

Supplementary Figure 14 – Structural comparison of TDP-43 NTD with DIX domains.

(a) Electrophoretic mobility shift assay (EMSA) with recombinant wild type TDP-43 NTD shows specific (but low affinity) protein-DNA complex with $(TG)_6$ oligonucleotide compared to a random DNA oligo of similar length.

(a) Overlay of monomer structures of TDP-43 NTD (blue), Axin-1 DIX (magenta, PDB ID 1WSP), Ccd-1 DIX (green, PDB ID 3PZ7) and Ubiquitin (orange, PDB ID 1TBE).

(b-d) Comparison of helical structures of Axin-1 DIX (a, PDB ID 1WSP), Dvl-2-DIX (b, PDB ID 4WIP) with TDP-43 NTD (c). The repeat length and the filament diameter for each structure are depicted on the side and bottom of the structures respectively.

Supplementary Table 1 – List of Antibodies

Antibody name	Company (Catalog number)	Dilution
Anti-human TDP-43 (6H6)	Proteintech (60019-2-Ig)	1:5000 (WB)
Anti-TDP-43 (3H8)	Antibodies-online (AB1N487384)	1:1000 (WB), 1:500 (IF)
Anti-TDP-43	Bethyl (A303-223A)	1:10,000 (WB)
Anti-TDP-43	Proteintech (18280-1-AP)	1:500 (IF)
Anti-phosphoTDP-43	Cosmo Bio (TIP-PTD-M01)	1:500 (IF)
Anti-phosphoTDP-43	Cosmo Bio (TIP-PTD-M01)	1:1000 (WB)
Anti-SOD1	Lifespan biosciences (AD1-SOD-100-D)	1:5000 (WB)
Anti-GAPDH	Abcam (ab8245)	1:5000 (WB)
Anti-Histone H3	Abcam (ab1791)	1:5000 (WB)
Anti-Actin	Sigma-Aldrich (A5441)	1:10,000 (WB)
Anti-GFP-FITC	Rockland antibodies (600-102-215)	1:500 (IF)
Anti-TIA1	Santa Cruz (sc-1751)	1:500 (IF)
Anti-hnRNPA1	Abcam (9H10)	1:1000 (WB)
Anti-FUS	Bethyl (A300-293A)	1:5000 (WB)

Supplementary Table 2 – List of primers for alternative splicing events

Gene name	Primer sequence	Annealing temperature
Mouse *Sort1*	*Forward primer* CAGGAGACAAATGCCAAGGT<br>*Reverse primer* TGGCCAGGATAATAGGGACA	58°C
Mouse *Dnjac5*	*Forward primer* CTCTATGTGGCGGAGCAGTT<br>*Reverse primer* GCTGTATGACGATCGGTGTG	58°C
Mouse *Eif4h*	*Forward primer* ACTTCGTGTGGACATTGCAG<br>*Reverse primer* CCCCCTACCCCCTAAGAAGT	58°C
Mouse *Sema3F*	*Forward primer* ACAACCCCATGTGCACCTAT<br>*Reverse primer* AGGGATGAGCTCAGCATGTA	58°C
Mouse *Poldip3*	*Forward primer* CATTGGGACTGTAACCCCAG<br>*Reverse primer* TGCAAACTTCATCTGCTTGG	58°C
Mouse *Tsn*	*Forward primer* CCCGAGAGGCTGTTACAGAG<br>*Reverse primer* CCTCGGATGGAAAGGTCATA	58°C
Mouse *Adpn2*	*Forward primer* CCTGTGCAGAATCTTGACAACA<br>*Reverse primer* AGAAACATCTCCCCACGATGT	56°C
Mouse *App*	*Forward primer* CCACAACCACCACTGAGTCC<br>*Reverse primer* ATTCTCTCTCGGTGCTTGG	58°C

Primer3 (http://bioinfo.ut.ee/primer3/) was used to design specific primer sequences

Supplementary Table 3 – List of primers for cloning and site-directed mutagenesis

TDP-43 mutation	Primer sequence (5'-3')
E17AE21A	*Forward primer* GAACGATGAGCCCATTGCAATACCATCGGCAGACGATGGGACGGTGC
	Reverse primer GCACCGTCCCATCGTCTGCCGATGGTATTGCAATGGGCTCATCGTTC
E14A	*Forward primer* CCGAAGATGAGAACGATGCGCCCATTGCAATACCATCGGCAGAC
	Reverse primer GTCTGCCGATGGTATTGCAATGGGCGCATCGTTCTCATCTTCGG
Q34A	*Forward primer* CTCTCCACGGTTACAGCCGCGTTTCCAGGGGCGTGTGGG
	Reverse primer CCCACACGCCCCTGGAAACGCGGCTGTAACCGTGGAGAG
R52AR55A	*Forward primer* CCAGTGTCTCAGTGTATGGCAGGTGTCGCGCTGGTAGAAGGAATTCTGC
	Reverse primer GCAGAATTCCTTCTACCAGCGCGACACCTGCCATACACTGAGACACTGG
N12A	*Forward primer* CGGGTAACCGAAGATGAGGCCGATGAGCCCATTGAAATACCATCG
	Reverse primer CGATGGTATTTCAATGGGCTCATCGGCCTCATCTTCGGTTACCCG
E58A	*Forward primer* GAGGTGTCCGGCTGGTAGCAGGAATTCTGCATGCCCCAGATGC
	Reverse primer GCATCTGGGGCATGCAGAATTCCTGCTACCAGCCGGACACCTC
ΔNTD	*Forward primer* ATGGATGAGACAGATGCTTCATCAGCAGTGAAAGTG
	Reverse primer CAAGTCCTCTTCAGAAATGAGCTTTTGCTCCATGG
pET28a(+)-NTD	*Forward primer* GACGGCTAGCTCTGAATATATTCGGGTAAC
	Reverse primer GACGAAGCTTCTAATCTTTTGGATAGTTGAC
